# Recent Advances of MSCs in Renal IRI: From Injury to Renal Fibrosis

**DOI:** 10.3390/bioengineering11050432

**Published:** 2024-04-27

**Authors:** Xinhao Niu, Xiaoqing Xu, Cuidi Xu, Yin Celeste Cheuk, Ruiming Rong

**Affiliations:** 1 Department of Urology, Zhongshan Hospital, Fudan University, Shanghai 200032, China; 2 Shanghai Key Laboratory of Organ Transplantation, Shanghai 200032, China

**Keywords:** mesenchymal stem cells, renal ischemia-reperfusion injury, renal fibrosis, myofibroblast, metabolic reprogramming, immunomodulation

## Abstract

Renal fibrosis is a pathological endpoint of maladaptation after ischemia-reperfusion injury (IRI), and despite many attempts, no good treatment has been achieved so far. At the core of renal fibrosis is the differentiation of various types of cells into myofibroblasts. MSCs were once thought to play a protective role after renal IRI. However, growing evidence suggests that MSCs have a two-sided nature. In spite of their protective role, in maladaptive situations, MSCs start to differentiate towards myofibroblasts, increasing the myofibroblast pool and promoting renal fibrosis. Following renal IRI, it has been observed that Bone Marrow-Derived Mesenchymal Stem Cells (BM-MSCs) and Renal Resident Mesenchymal Stem Cells (RR-MSCs) play important roles. This review presents evidence supporting their involvement, discusses their potential mechanisms of action, and suggests several new targets for future research.

## 1. Introduction

Acute kidney injury (AKI) is a major problem challenging global public health and occurs in approximately 13 million people worldwide each year, posing a large medical burden to families and society [1]. Because the mechanisms behind kidney injury are complex, and the process is mostly irreversible, the literature reports that less than 50% of patients are cured with pharmacological or short-term renal replacement therapy (RRT) [2]. RRT refers to medical strategies such as dialysis, employed to replace the waste-filtering functions of a failing kidney. The kidneys that are not properly cured eventually go to the pathological endpoint of renal fibrosis [3] and develop chronic renal insufficiency and even end-stage renal disease. Ischemia-reperfusion injury (IRI) is the primary surgical cause of AKI, and the incidence of AKI in renal transplant patients can be as high as 11.3–17.1% [4]. Therefore, exploring the cellular mechanisms of renal fibrosis development after renal IRI and finding the targets of intervention have become hot topics.

Accumulated evidence has identified myofibroblasts as essential cells in kidney fibrosis [5]. After renal IRI, various pathological processes, such as oxidative stress, hemodynamic alterations, renal tubular injury, endothelial microvascular system malfunction, and inflammatory immune response can lead to renal cell damage and the release of a series of inflammatory mediators that induce an inflammatory response, further aggravating the degree of cell damage, while causing a variety of cells to differentiate into myofibroblasts [6,7]. The excessive collagen fibers secreted by myofibroblasts are not easily degraded, and the accumulation and deposition of large amounts of extracellular matrix collagen destroy the kidney structure and eventually form scar tissue, a process that irreversibly causes a decrease in renal function [8].

Mesenchymal stem cells (MSCs) are a type of multipotent stem cell from the mesenchyme that has been proven to be derived from almost all tissues’ adventitial progenitor cells and pericytes [9,10,11]. MSCs exist in the bone marrow, adipose tissue, cord cells, molar cells, amniotic fluid, placenta, and other tissues, and have shown potential for cellular therapeutic applications in acute and chronic renal injury, diabetic renal fibrosis, and other kidney diseases [12,13]. However, MSCs are not always beneficial [14]. It has been confirmed that under the stimulation of pro-fibrotic factors such as transforming growth factor-beta 1 (TGF-β1), MSCs can differentiate into myofibroblasts, thereby losing their renal protective function and increasing the pool of myofibroblasts in the organ, accelerating the process of renal fibrosis [15,16,17]. This review specifically targets the elucidation of MSCs’ mechanisms of action in the context of renal IRI, evaluates their two-sided nature of both alleviating damage and promoting renal fibrosis, and identifies gaps in current knowledge that future research must address to fully harness MSCs’ therapeutic potential.

In this narrative review, we will discuss the role and characteristics of MSCs in the different periods from renal IRI to renal fibrosis, aiming to provide overall and new insights into the prevention of renal fibrosis.

## 2. Method

To compile an exhaustive overview, in the preparation of this review, we systematically searched across PubMed, Scopus, and Web of Science databases using the keywords ‘mesenchymal stem cells’ or ‘MSCs’, ‘Ischemia-reperfusion injury’ or ‘IRI’, ‘Fibrosis’, ‘renal’ or ‘kidney’. The search was focused on articles published from 2000 to 2023. Inclusion criteria were animal studies, clinical trials, and reviews, whereas conference abstracts and editorial articles were excluded. A total of 196 articles were initially identified, and after screening for relevance and quality based on predefined criteria, 70 articles were included in this review.

## 3. The Dual Nature of MSCs in Renal IRI

In the past decade, as research in the field of stem cells has progressed, the role of mesenchymal stem cells (MSCs) in the transition from acute kidney injury to renal fibrosis has been gradually uncovered. MSCs are multipotent stem cells derived from the mesenchyme. The International Cell Therapy Association has established the minimum standard for human MSCs definition: must be adherent cells; must exhibit a three-lineage differentiation potential that can differentiate into osteoblasts, adipocytes, and chondrocytes; must express certain surface patterns of CD105, CD73, and CD90, while lacking CD45, CD34, CD14, CD11b, or CD79a or the expression of CD19, as well as HLA-DR [18]. MSCs not only have strong plasticity and tissue repair abilities but also can secrete a variety of cytokines and regulate the immune response [19,20]. Numerous animal-based studies have demonstrated that MSCs injection therapy or the use of MSCs exosomes in the early stages of renal IRI can effectively reduce the extent of kidney injury and prevent the development of renal fibrosis [21,22,23]. In addition, Bone Marrow-Derived MSCs (BM-MSCs) recruited to the kidney via the peripheral blood in response to injury signals can also exert renoprotective effects by inhibiting inflammatory cell infiltration in the kidney and promoting an increase in immunomodulatory cells. BM-MSCs can exert renoprotective effects by secreting indoleamine 2,3 dioxygenase (IDO) and other immunosuppressive factors to promote the proliferation of regulatory T cells and inhibit the pro-inflammatory effects of helper T cells 17(Th17), thereby reducing renal tubular epithelial cell injury [24].

**Figure 1:**
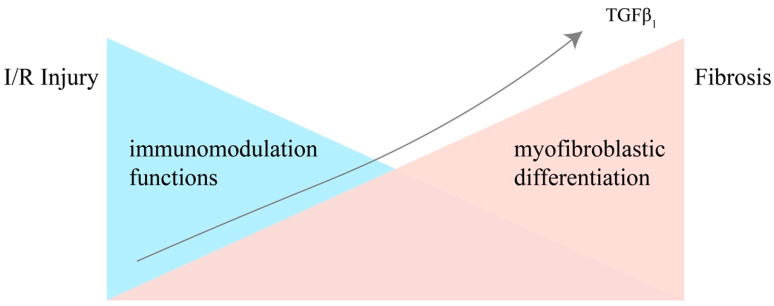
The immunomodulative functions and differentiating potential of MSCs in the process from IRI to renal fibrosis, under the stimulation of accumulating TGF-β1. Injury leads to a storm of inflammatory cytokines in the kidney, and the more inflammatory cytokines are secreted, the stronger the MSCs’ immunomodulatory capacity becomes. However, the immunomodulatory abilities of MSCs are negatively correlated with their differentiation potential. Under the stimulation of accumulating TGF-β1 secreted by the injured epithelial cells and immune cells, MSCs gradually lose the ability of immunomodulation and differentiate into myofibroblasts.

Paradoxically, the role of MSCs is not always protective. Undifferentiated MSCs can exert renoprotective functions through reparative cytokines and immunomodulation. However, MSCs also have an extremely strong proliferative and multidirectional differentiation potential, which could be a double-edged sword. Some studies have confirmed that under the stimulation of pro-fibrotic factors such as TGF-β1, MSCs can differentiate into myofibroblasts, thereby losing their renoprotective function and increasing the pool of myofibroblasts in the organ, accelerating the process of renal fibrosis [15,16,17,25] (Figure 1). Myofibroblasts are one of the participants in the tissue repair process, and in the physiological condition they are activated in response to injury signals to produce an extracellular matrix enriched with type I and type III collagen to maintain the structural and functional integrity of tissues, but the excessive accumulation and overactivation of myofibroblasts has been recognized in recent years as an important step of the formation of renal fibrosis [8]. In the previous literature reports, Le Bleu et al. found that only about 35% of renal myofibroblasts are derived from circulating BM-derived cells, and the other 65% are from renal-resident cells [26], while Kramann et al. demonstrated that a great proportion of renal myofibroblasts are derived from renal-resident MSCs (RR-MSCs) [27]. Therefore, RR-MSCs have recently been considered as an important source of myofibroblasts-driving renal fibrosis.

Quan Zhuang et al. described four consecutive phases from acute kidney injury to renal fibrosis [12], namely the acute inflammatory phase, the prophase of fibrosis (signaling pathway transduction phase), the fibrosis formation phase, and the final loss of renal function, with MSCs playing an active role in the first two phases. Through our literature review, we found that MSCs may exert different effects in different phases. Therefore, we will explore this topic across different stages.

## 4. MSCs in the Early Stage of Injury

Within the first few days after the onset of IRI, a variety of innate immune cells, including M1-type macrophages, neutrophils, mast cells, dendritic cells, and natural killer (NK) cells, and a variety of adaptive immune cells, such as T cells and B cells, all accumulate at the site of inflammation and release a large number of inflammatory mediators, such as interleukin-1 beta (IL-1β), tumor necrosis factor-alpha (TNF-α), and interleukin-6 (IL-6), which together form the inflammatory response microenvironment [28]. Under the stimulation of inflammatory mediators, RR-MSCs are activated, and BM-MSCs in the peripheral blood also converge to the site of inflammation. According to the literature, RR-MSCs and BM-MSCs do not differ significantly in their functions in the early stages of inflammation, playing a regulatory role in the inflammatory microenvironment by producing a variety of cytokines. It has been shown that MSCs can inhibit the proliferation and activity of T cells in their microenvironment by producing Inductible Nitric Oxide Synthase (iNOS) or IDO [29,30,31]. MSCs can also induce macrophage polarization from a pro-inflammatory M1 phenotype to an anti-inflammatory M2 phenotype by producing spermidine [32]. In addition, the immunosuppressive function of MSCs also acts through TGF-β1, IL-6, hepatocyte growth factor (HGF), leukemia inhibitory factor (LIF), anti-inflammatory mediator prostaglandin E2 (PGE2), tumor necrosis factor-stimulated gene 6 protein (TSG6), heme oxygenase 1 (HO1), and galactocortin [33,34,35,36]. These cytokines inhibit the proliferation and function of pro-inflammatory immune cells, such as helper T (TH) cells, pro-inflammatory macrophages, neutrophils, natural killer (NK) cells and B cells, and enhance the proliferation and function of anti-inflammatory immune cells, such as M2 macrophages and regulatory T (Treg) cells, while proliferating anti-inflammatory immune cells can further inhibit the activity and function of pro-inflammatory immune cells. The proliferating anti-inflammatory immune cells can further suppress the activity and function of pro-inflammatory immune cells, thereby inhibiting the progression of inflammation and promoting tissue repair. In addition, microRNAs (miRNAs) in exogenous BM-MSCs exosomes, such as miR-34c-5p, have been shown to have immunosuppressive effects in acute inflammation due to acute kidney injury by inhibiting the core functions of various proteins [37]. In addition to reducing the early inflammatory response through the above-mentioned immunosuppressive effects, MSCs can also promote renal neovascularization and tissue repair processes through the secretion of growth factors, such as epidermal growth factor (EGF), HGF, insulin-like growth factor (IGF), and vascular endothelial growth factor (VEGF) [38,39,40]. They play a critical role in angiogenesis. It should be noted that there are several isoforms of VEGF, each having distinct functions. In this context, we specifically focus on VEGF-A due to its prominent role in the regulation of endothelial cell proliferation and vascular permeability. In a word, during the acute inflammatory phase, both RR-MSCs and circulating BM-MSCs play a role as “firefighters” by reducing renal injury through negative immunomodulation, inhibiting inflammatory factor expression, promoting vascular regeneration, and facilitating tissue repair. While a consensus emerges on the protective role of MSCs against renal IRI, variability in outcomes across studies highlights the influence of experimental conditions, sources of MSCs, and timing of administration. Furthermore, the underlying mechanisms of MSCs’ action remain inadequately understood, signaling a need for in-depth mechanistic studies.

## 5. MSCs in the Progression of Renal Fibrosis

**Figure 2:**
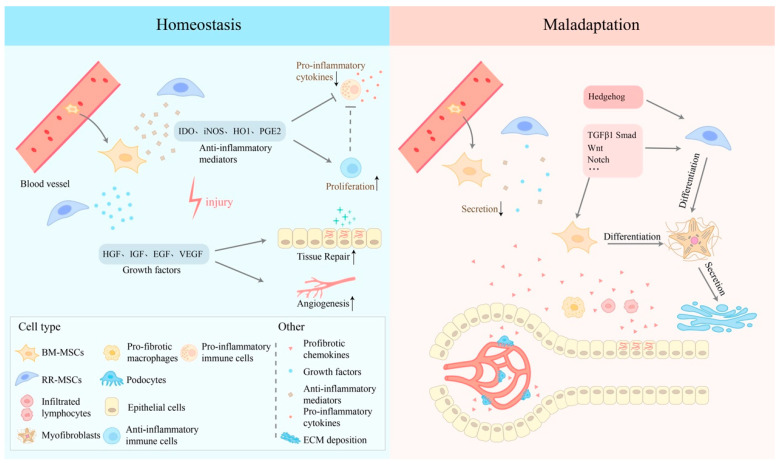
Mechanisms of the two-sided nature of MSCs in homeostasis or in maladaptive conditions after acute I/R injury. After the onset of ischemia-reperfusion injury, under the stimulation of inflammatory mediators, RR-MSCs are activated, and BM-MSCs in the peripheral blood converge to the site of inflammation. Anti-inflammatory mediators, such as iNOS (mouse) or IDO (human), prostaglandin E2 (PGE2), heme oxygenase 1 (HO1) etc., are released by both kinds of MSCs, along with growth factors, including epidermal growth factor (EGF), hepatocyte growth factor (HGF), insulin-like growth factor (IGF), and vascular endothelial growth factor (VEGF) etc. These mediators can inhibit the pro-inflammatory immune cells, reducing the level of pro-inflammatory cytokines and promoting tissue repair and angiogenesis. However, in the maladaptive condition, TGF-β1 and other profibrotic chemokines secreted by injured epithelial cells, podocytes, and profibrotic immune cells can inhibit the ability of immunomodulation of MSCs and drive myofibroblastic differentiation, aggravating ECM collagen deposition.

**Figure 3:**
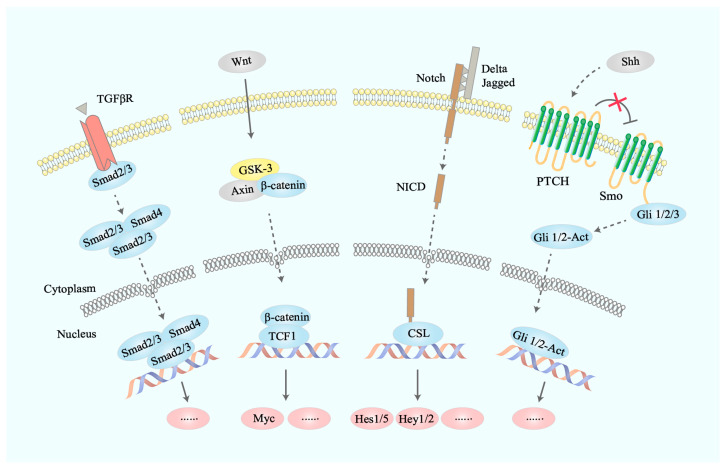
Comprehensive diagram of the involved signal pathways.

After renal IRI occurs, damaged renal cells and various immune cells in the inflammatory microenvironment can continue to produce cytokines such as TGF-β1 for early repair, but these cytokines may also have pro-fibrotic effects. It has been shown that in the early stage, BM-MSCs circulating to the kidney and RR-MSCs can still secrete IDO and other immunomodulatory factors to resist the process of renal fibrosis mediated by small doses of TGF-β1; when the injury continues to worsen and the microenvironmental inflammation increases and exceeds the regulatory capacity of MSCs, a large number of MSCs will differentiate into myofibroblasts under the stimulation of large amounts of TGF-β1 and other pro-fibrotic factors [15,16,41] (Figure 2). The differentiated MSCs will lose their immunomodulatory function, and MSCs-derived myofibroblasts will further secrete more pro-fibrotic factors. This process is accompanied by the activation of multiple pro-fibrotic signaling pathways, such as TGF-β1/Smad, Notch, Wnt, Hedgehog, etc. Consequently, renal injury progresses into the prophase of fibrosis [12] (Figure 3). 

## 6. Signal Pathways

### 6.1. TGF-β1/Smad Pathway

Over the past few decades, TGF-β1 has been used to induce myofibroblastic differentiation because TGF-β1 is a common link in the process of fibrosis and a major factor promoting fibrosis [42]. The TGF-β1/Smad pathway is considered one of the most classical fibrotic signaling pathways and also plays a key role in the progression of renal fibrosis, which was identified by researchers as a therapeutic target for renal fibrosis back in the 1990s [16]. Under physiological conditions, low concentrations of TGF-β1 are present in the extracellular matrix together with latency-associated peptides (LAPs) and latency TGF-β binding proteins (LTBPs), which together form the large latency complex (LLC) [43]. When IRI occurs, on the one hand, LAP in the extracellular matrix is hydrolytically cleaved, leading to the release of TGF-β1 from the LLC and its binding to the receptor for its effect. On the other hand, immune cells recruited to the site of injury can also directly secrete large amounts of TGF-β1 to bind directly to the receptor. Subsequently, the activated receptor is able to bind and phosphorylate the Smad protein, a central regulator of the classical TGF-β1 signaling pathway. There are three classes of Smad proteins: regulatory (R)-Smads (Smad2 and Smad3), coactivators (Co)-Smads (Smad and Smad4) and inhibitory (I)-Smads (Smad6 and Smad7). The main effector in the fibrosis signaling pathway is the R-Smad family, especially Smad3 [44,45]. TGF-β1 signaling leading to Smad3 activation plays an important role in fibrosis caused by acute kidney injury. On the one hand, TGF-β1 can act directly on RR-MSCs and BM-MSCs, as well as other primitive cells, such as fibroblasts and pericytes, prompting their differentiation to myofibroblasts. It also promotes epithelial-mesenchymal transition (EMT) in epithelial cells, further increasing the pool of myofibroblasts in diseased kidneys. Additionally, the presence of TGF-β1 stimulates myofibroblasts to secrete large amounts of collagen fibers into the extracellular matrix [16,41]; on the other hand, there may be an interaction of Smad3 with extracellular signal-regulated kinase (ERK) and Janus kinase/signal transducer and activator of transcription (JAK-STAT), exacerbating collagen accumulation in the ECM and inhibiting its breakdown, thus exacerbating fibrosis [46].

The TGF-β1/Smad pathway exacerbates fibrosis not only by promoting the activation and differentiation of primitive cells such as fibroblasts and pericytes into myofibroblasts and, but also by promoting the myofibroblastic differentiation of RR-MSCs and circulating BM-MSCs. These cells, which originally act as “firefighters”, lose their original protective function and participate in the process of promoting fibrosis.

### 6.2. Notch Signaling Pathway

The Notch signaling pathway is a phylogenetically conserved intercellular communication mechanism that distinguishes adjacent cells through the asymmetric expression of pathway ligands and receptors with a variety of distinct functions, including cell fate determination, cell lineage specification, and cell lineage stabilization, and has a major role in proximal tubule cell and podocyte differentiation during kidney development in physiological states [47]. In healthy adult kidneys, Notch signaling activity is low and the increased Notch receptor expression is usually associated with epithelial dedifferentiation, myofibroblast activation, stromal deposition, and inflammatory responses [48]. The mechanisms by which Notch signaling promotes the development of fibrosis are not fully understood, and studies have demonstrated a role for Notch activation in processes associated with renal fibrosis, such as podocyte apoptosis, fibroblast activation, etc. [49].

### 6.3. Wnt/β-Catenin-Signaling Pathway

The Wnt/β-catenin pathway was once thought to be only a cancer-related signaling pathway [50], but it was later found to be also an essential signaling pathway involved in renal tubular epithelial repair after AKI. However, Wnt/β-catenin signaling also has a promotional role in renal fibrosis by promoting the conversion of mesenchymal fibroblasts and pericytes to myofibroblasts [51]. Wnt proteins are a class of secreted lipid-modified glycoproteins, while their downstream β-catenin proteins are key mediators that, at a steady state, are regulated by glycogen synthase kinase 3β (GSK3β), scaffolding protein axis protein, casein kinase 1α, and adenomatous colonic polyp protein, which phosphorylates the free N-terminal end of β-catenin protein to degrade it ubiquitously in the cytoplasm. In the presence of Wnt proteins, the non-phosphorylated β-catenin protein accumulates in the cytoplasm and is transferred to the nucleus to regulate Wnt target genes [52,53]. After the onset of AKI, this process is activated to promote the differentiation of fibroblasts and pericytes in the mesenchyme towards myofibroblasts, thus increasing the myofibroblast pool and contributing to renal fibrosis [51]. In addition, a study reported Notch and Wnt pathway interactions in intestinal tissue-resident stem cells, with researchers observing high Notch and Wnt activity in the intestinal Lgr5+ stem cell populations [54]. This interaction may be related to the transit expansion and lineage determination of intestinal cells [55]. A similar mechanism might also exist in RR-MSCs, but has not yet been reported in the literature.

### 6.4. Hedgehog Pathway

The Hedgehog (Hh) pathway is also one of the important developmental signaling pathways in the physiological state and plays an important role in the development of the mammalian embryo, including ventralization of the neural tube, organ formation, and growth and development of the limbs and face [46]. Three Hedgehog proteins have been identified in mammals: Sonic Hedgehog (Shh), Desert Hedgehog (Dhh), and Indian Hedgehog (Ihh), with Shh and Ihh playing a major role in the process of fibrosis. The Hh family of proteins is known as a bridge between epithelial and mesenchymal communication. These secretory proteins act in an autocrine or paracrine manner by binding to Patched1, a membrane receptor on target cells, to activate smooth transmembrane proteins (SMO) on the cytosol that are inhibited by Patched1. SMO activation leads to an intracellular signaling cascade that drives activation and nuclear translocation of Gli family transcription factors. In mammals, three members of the Gli transcription factor family are present: Gli1 and Gli2 (both activators) and Gli3 (major repressors) [56]. In the kidney, expression of Shh and Ihh is barely detectable in the physiological state, but after renal IRI, both Shh and Ihh are abundantly expressed by damaged tubular epithelial cells, which in turn cause an SMO activation signaling cascade that ultimately drives activation of Gli proteins and their nuclear translocation, leading to expression of Hh target genes such as Gli1, resulting in proliferation and activation. It has also been reported that the Hedgehog signaling pathway can interact with TGF-β1, Wnt, and Notch pathways during the progression of renal fibrosis, causing further aggravation of fibrosis [57].

## 7. Promising Targets

Since the process of pathological collagen deposition in the renal interstitium is almost irreversible, once they are produced, the acute inflammatory phase and the prophase of fibrosis are the most critical times for intervention. In this case, only by timely intervention before the collagen fibers are actually produced in the extracellular matrix can we possibly avoid irreversible renal damage. Thus, in this part we aim to discuss several possible targets in the process of myofibroblastic differentiation of MSCs.

Gli proteins: In the above mentioned Hedgehog pathway, renal resident Gli1+ MSCs may play an important role, as these cells also express Gli1 transcription factors, making them target cells of the Hh signaling pathway. Consequently, they eventually differentiate into myofibroblasts in response to the signaling cascade [58,59]. This may also be the reason why in some studies, RR-MSCs are more likely to differentiate into myofibroblasts compared to BM-MSCs. In fact, although no direct reports were seen on the extent of the contribution of renal tissue-resident Gli1+ MSC to the myofibroblast pool after renal IRI, it has been shown that Gli1+ MSC-like cells, which account for less than 0.2% of the total PDGFRβ+ renal cell population, are reported to be the source of approximately 50% of myofibroblasts in a murine model of unilateral ureteral obstruction kidney injury and are considered to be the most predominant cell population involved in renal fibrosis [27]. Thus, Gli proteins might be potential therapeutic targets in kidney fibrosis. However, MSCs are not the only cells that express Gli1 in vivo. There are numerous cells expressing Gli1 and playing an important role in maintaining homeostasis. A study observed a reduction in renal fibrosis induced by the Hh signaling pathway after depletion of Gli1+ cells, but it also led to concomitant capillary thinning and increased renal tubular damage, which also suggests that Gli1 cannot be used indiscriminately as an intervention target to reduce fibrosis in future studies [60]. 

MSCs differentiation: In light of the two-sided nature of MSCs, new possible targets should be explored from the process of myofibroblastic differentiation of MSCs, aiming to prevent their differentiation and thus preserve their immunomodulatory abilities. Cell differentiation is a complex process that involves many epigenetic, transcriptional, and metabolic alterations. To date, no studies have been conducted directly intervening in the differentiation of MSCs into myofibroblasts. However, there is growing evidence that metabolic alterations are not only an essential source of energy for cell differentiation, but also that a variety of metabolites also act as substrates for enzymes that can post-translationally modify transcription factors and histones. These modifications, in turn, control gene transcription and regulate cell differentiation [61,62,63]. Thus, metabolic reprogramming is currently considered a key direct regulator of cell differentiation. In an aerobic environment, most of the intracellular pyruvate enters the mitochondria to participate in the tricarboxylic acid cycle (TCA cycle) and undergoes oxidative phosphorylation to produce ATP, whereas in the hypoxic or anaerobic state, pyruvate is directly converted to lactate by glycolysis to produce energy [64]. However, some cells maintain low levels of oxidative phosphorylation, even under aerobic conditions, preferentially using glycolysis for most of their energy. This switch in the cell’s primary energy supply from oxidative phosphorylation to glycolysis is called metabolic reprogramming. This phenomenon was first proposed by Warburg and is therefore known as the Warburg effect [65]. The Warburg effect was initially discovered in tumor cells, but later it was gradually found in non-malignant cells and had a direct effect on cell differentiation. During the differentiation of cortical neuronal cells, Agostini et al. observed changes in cellular mitochondrial quantity, morphology, and function, accompanied by upregulation of mitochondrial transcription factor A (TFAM) and peroxisome proliferators-activated receptor γ coactivator 1α (PGC-1α). At the same time, glucose uptake, glucose transporter 3 (GLUT3) expression, and glutamate-glutamine metabolism were increased. Therefore, they concluded that metabolic reprogramming is closely related to neuronal cell differentiation and identified the PI3K-Akt-mTOR pathway as a key mediator of neuronal energy metabolism [63]. Furthermore, similar findings were found in renal podocytes, where investigators found that differentiated podocytes exhibited higher rates of acidification in their cultures. Metabolomic analysis revealed significant differences between undifferentiated and differentiated podocytes in glycometabolism products, as well as elevated expression of key enzymes involved in glycometabolism and GLUT. These findings indicate elevated levels of aerobic glycolysis in differentiated podocytes compared to undifferentiated podocytes [61]. It has also been reported that there is an elevated proportion of glycolysis and reduced oxidative phosphorylation during the chondrogenic differentiation of MSCs [66]. It could be hypothesized that, like many progenitor cells, MSCs are predominantly energized by oxidative phosphorylation to maintain their secretory and immunomodulatory functions under normal conditions, whereas an elevated proportion of glycolysis occurs during their differentiation. This mechanism may also account for the loss of the secretory function of MSCs during their differentiation to myofibroblasts under the stimulation of TGF-β1. Therefore, targeting the metabolic reprogramming of MSCs to modulate their myofibroblastic differentiation may be a new promising research direction in the future.

## 8. Discussion

Myofibroblasts are now widely considered to be the core of renal fibrosis, and almost all pathological renal fibrosis can be attributed to excessive deposition of collagen fibers in the extracellular matrix produced by myofibroblasts [12]. The origin of myofibroblasts mainly involves the transformation of fibroblasts in the renal mesenchyme, differentiation of pericytes and podocytes, epithelial-mesenchymal transition (EMT), endothelial-mesenchymal transition (EndoMT), as well as myofibroblastic differentiation of BM-MSCs and RR-MSCs [7,26]. However, the exact percentage of each source contributing to the myofibroblast pool is still controversial. It was reported that EMT was the main source of myofibroblasts, but most of this conclusion came from in vitro experiments and was not fully validated in vivo [67,68,69]. Some researchers claim that the proportions consist of fibroblasts (50%), bone marrow-derived cells (35%), endothelial cells (10%), and epithelial cells (5%) [17,41]. However, it has also been demonstrated that renal-resident Gli1+ MSC-like cells alone can contribute approximately 50% of myofibroblasts, although they only represent 0.2% of the total PDGFRβ+ (a mesenchymal marker that identifies interstitial cells) renal cell population [27]. This topic may remain controversial because the discrepancies in these findings might be related to differences in animal models, as well as to differences in detection methods. For now, it remains unclear whether MSCs can induce other cells to differentiate into myofibroblasts. However, we chose MSCs as the focus of this review because, on the one hand, they have been reported in the literature as one of the important sources of myofibroblasts in the kidney, and on the other hand, because of their two-sided nature, they lose immunomodulatory capacity during myofibroblastic differentiation.

After the onset of renal IRI, the involvement of BM-MSCs and RR-MSCs has been reported, but the existing literature did not compare the specific contribution of both to renal fibrosis. We speculate that RR-MSCs contribute more to the myofibroblast pool. On the one hand, this may be because they undergo ischemia-reperfusion in the kidney, which may activate additional signaling pathways. On the other hand, some unique proteins in RR-MSCs, such as Gli1, make them subject to exclusive signaling pathway regulation. In addition, the markers specific to RR-MSCs are still controversial, with some studies suggesting Nestin to be the identifying marker [70], some believing that it is Sca-1 [71,72], and others considering it to be Gli1 [27]. In future studies, an attempt could be made to find a more proper marker to better distinguish the two subsets of MSCs and analyze their role in renal fibrosis separately, which may lead to more meaningful findings.

While the mechanisms through which MSCs contribute to the myofibroblast pool, primarily through myofibroblastic differentiation induced by pro-fibrotic factors such as TGF-β1, are increasingly understood, questions remain unclear regarding the potential of MSCs to directly induce the differentiation of other cell types into myofibroblasts. Given the fact that MSCs only represent a very low proportion of all renal interstitial cells, their number might not be sufficient to directly induce renal fibrosis. The capacity of MSCs to influence distant cellular populations suggests complex intercellular communication that may facilitate the fibrotic process, but the way MSCs induce other kind of cells to differentiate into myofibroblasts has not yet been explored. Further studies are required to elucidate the full spectrum of MSCs-induced cellular differentiation within the fibrotic kidney.

Regulating the differentiation of MSCs into myofibroblasts via metabolic reprogramming is a novel topic that has not yet been reported. This may provide new ideas for future studies.

## Data Availability

Not applicable.
